# An Objective Assessment of Neuromotor Control Using a Smartphone App After Repeated Subconcussive Blast Exposure

**DOI:** 10.3390/s24217064

**Published:** 2024-11-02

**Authors:** Charlend K. Howard, Masahiro Yamada, Marcia Dovel, Rie Leverett, Alexander Hill, Kenneth A. Manlapaz, David O. Keyser, Rene S. Hernandez, Sheilah S. Rowe, Walter S. Carr, Michael J. Roy, Christopher K. Rhea

**Affiliations:** 1 Ellmer College of Health Sciences, Old Dominion University, Norfolk, VA 23529, USA; c1howard@odu.edu; 2 Department of Kinesiology, Whittier College, Whittier, CA 90602, USA; myamada@whittier.edu; 3 Military Traumatic Brain Injury Initiative (MTBI2), Uniformed Services University, Bethesda, MD 20814, USA; marcia.dovel.ctr@usuhs.edu (M.D.); rie.leverett.ctr@usuhs.edu (R.L.); alexander.hill.ctr@usuhs.edu (A.H.); kenneth-andres.manlapaz.ctr@usuhs.edu (K.A.M.); david.keyser@usuhs.edu (D.O.K.); rene.hernandez.ctr@usuhs.edu (R.S.H.); sheilah.rowe.ctr@usuhs.edu (S.S.R.); michael.roy@usuhs.edu (M.J.R.); 4 Henry M. Jackson Foundation for the Advancement of Military Medicine, Bethesda, MD 20817, USA; 5 Department of Military and Emergency Medicine, Uniformed Services University, Bethesda, MD 20841, USA; 6 Walter Reed Army Institute of Research, Silver Spring, MD 20910, USA; walter.s.carr.civ@health.mil; 7 Department of Medicine, Uniformed Services University, Bethesda, MD 20814, USA

**Keywords:** neuromotor, balance, military, blast exposure, subconcussive, smartphone app

## Abstract

Subconcussive blast exposure has been shown to alter neurological functioning. However, the extent to which neurological dysfunction persists after blast exposure is unknown. This longitudinal study examined the potential short- and long-term effects of repeated subconcussive blast exposure on neuromotor performance from heavy weapons training in military personnel. A total of 214 participants were assessed; 137 were exposed to repeated subconcussive blasts and 77 were not exposed to blasts (controls). Participants completed a short stepping-in-place task while an Android smartphone app placed on their thigh recorded movement kinematics. We showed acute suppression of neuromotor variability 6 h after subconcussive blast exposure, followed by a rebound to levels not different from baseline at the 72 h, 2-week, and 3-month post-tests. It is postulated that this suppression of neuromotor variability results from a reduction in the functional degrees of freedom from the subconcussive neurological insult. It is important to note that this change in behavior is short-lived, with a return to pre-blast exposure movement kinematics within 72 h.

## 1. Introduction

While traumatic brain injuries (TBIs)—inclusive of concussions—have garnered increased attention over the years, there has been a growing concern regarding subconcussive head trauma [1,2,3,4,5]. Unlike TBIs, subconcussive head trauma does not result in a loss of consciousness but may lead to symptoms akin to those of a TBI [6]. One form of subconcussive head trauma among military populations is exposure to occupational blast overpressure, where a shock wave is coupled with a rapid rise in atmospheric pressure. For instance, breachers in the military deploy explosives in close proximity to expedite their movement. Many of these exposures occur at a purported subclinical level, meaning there is no diagnosable head injury resulting from the blast exposure. However, it has been shown that even subclinical blast exposure can impair cognitive performance and biological markers of neurotrauma can increase in the short term with potential longer-term consequences [2,4,5,7,8,9,10]. 

Most studies that have examined the neurological consequences of repeated subconcussive blast exposure (RSCBE) have utilized a between-subjects design and/or tracked neurological assessments for a relatively short period of time. For example, a study conducted by Tate et al. examined the impacts of RSCBE in military personnel who underwent a 2-week breaching course [5]. The authors noted shifts in symptoms, biomarker levels, and neurocognitive impairments after completion of the breaching training, suggesting that military personnel may undergo deteriorating effects from cumulative low-level blast exposure. While the results from Tate et al. [5] are important, the question of longer-term effects was unanswered. Until recently, there have been no within- and between-subjects longitudinal studies examining the neurological effects of RSCBE.

To meet this need, the INVestigating traIning assoCiated blasT pAthology (INVICTA) study was developed to assess the wide range of aspects of brain function, including immediate and delayed cognitive recall, neuromotor performance, audiologic and oculomotor function, cerebral blood flow, brain electrical activity, neuroimaging, and the blood biomarkers of neurotrauma [11]. INVICTA is an observational, prospective, longitudinal study featuring both within- and between-subject comparisons. This paper focuses on the neuromotor assessment portion of INVICTA. 

One way to examine the potential short- and long-term neurological consequences from subconcussive blast exposure is via a neuromotor test that assesses balance [7,12]. Balance assessments fall into objective or subjective categories [12]. Subjective tests, like the Balance Error Scoring System (BESS), rely on evaluators to judge performance across tasks [13], but their subjective nature raises concerns about their reliability and validity [14,15,16]. Conversely, objective tests like the Neurocom Sensory Organization Test (SOT) use sensors to measure neuromotor performance [17,18], reducing reliability issues but posing cost and space challenges, making field testing impractical. The Balance Tracking System (BTrackS) addresses cost and portability concerns [19,20]. However, BTrackS uses a static balance task, potentially missing subtle dysfunctional behavior detectable in dynamic tasks [21]. Thus, a previous barrier to the implementation of neuromotor assessment in field-based settings was the ability to collect dynamic balance performance in an objective manner (i.e., sensors) with a device that was portable and cost-effective. 

To address these concerns, an Android smartphone app—called AccWalker—was developed and utilizes the smartphone’s sensors (accelerometer, gyroscope, and magnetometer) to capture movement kinematics during a dynamic stepping-in-place task. Gait tasks have long been used to measure neuromotor performance in the context of aging [22], disease [23], and brain injury from head trauma [24]—inclusive of concussions [25]. Thus, it was logical to use a task like gait to examine neuromotor performance after subconcussive head trauma but with the acknowledgment that typical overground or treadmill tasks performed in a clinic or research laboratory were not good candidates for a field-based assessment due to equipment and/or space limitations. Previous work explored the utility of a stepping-in-place task as a surrogate for treadmill walking and showed similar temporal and spatial neuromotor performance between the tasks [26]. Importantly, variability in neuromotor performance has been shown to be an important construct when discriminating between clinical and non-clinical conditions [27,28,29]. This observation led to exploring variability in neuromotor performance—in particular, thigh-angle kinematics during a stepping-in-place task—in earlier studies with AccWalker and showed that the app and associated stepping-in-place protocol could discriminate between those with and without a history of concussion [30] and subconcussive exposure [7]. The app’s reliability and validity in objectively measuring thigh-angle kinematics have been reported [31]. Thus, AccWalker was implemented into the INVICTA protocol and reported in this paper. 

The purpose of this paper was to investigate the effects of RSCBE from heavy weapons training (HWT) on neuromotor performance in military personnel utilizing a longitudinal design. Utilizing a neuromotor/physiological variability framework [28,32,33,34,35,36,37,38], we hypothesized that participants going through HWT would exhibit neuromotor dysfunction indexed by lower variability in their movement during a dynamic balance task. The extent to which neuromotor variability remained suppressed over time was exploratory. 

## 2. Materials and Methods

### 2.1. Participants

A total of 214 active-duty military personnel were recruited for this study and all participants provided informed consent. From this population, 37 were Range Safety Officers (RSOs), 100 were Special Operators or trainees (SOs), 32 were naïve active-duty controls (Naïve Control), and 45 were in the training control group (Train Control). All participants signed an informed consent form presented by the principal investigator (PI) or other study staff. 

### 2.2. Design

INVICTA is a multi-site observational, prospective, longitudinal study featuring within- and between-subject comparisons to better understand the magnitude of RSCBE and document consequent cellular, neurocognitive, neuromotor, physiology, and functional changes. Participants are divided into two groups (blast-exposed vs. controls). Those in the exposed group (RSOs, SOs, SO Trainees; N = 137) went through HWT (i.e., firing Carl Gustave recoilless rifles, Light Anti-Armor Weapons, and AT-4 anti-tank rockets). Individuals were labeled as exposed if they received blast exposure ≥ 4 pounds per square inch (psi) as recorded by wearable pressure sensors (BlackBox Biometrics, Rochester, NY, USA). This magnitude aligns with the threshold presented in the “Interim Guidance for Managing Brain Health Risk from Blast Overpressure” memorandum released by the Assistant Secretary of Defense for Readiness on 4 November 2022. Control groups are participants who did not engage in HWT. Specifically, the naïve control group (N = 32) consisted of individuals who did not participate in training and were not on site, while the training control group (N = 45) consisted of individuals who did not complete HWT training but participated in other training exercises on site. The outcome assessments included in this paper were taken at baseline (prior to HWT for exposed participants), and at four time points after HWT: 6 h post, 72 h post, 2 weeks post, and 3 months post. This study design allowed for the identification of changes in dependent variables between the pre-exposure baseline and the post-exposure time points for between-group (exposed vs. control) and within-group comparisons. 

### 2.3. Materials

Android smartphones (Google Pixel, version 13.0) with the AccWalker app installed were used to obtain thigh-angle time series data during a stepping-in-place task recorded at 100.84 Hz, as described in Kuznetsov et al. [31]. The smartphone was attached to the participants’ thigh via an adjustable groin strap made of neoprene (McDavid USA, Woodridge, IL, USA) using a standard hook and loop material placed on the back of the smartphone and the strap.

### 2.4. Procedures

All testing was performed on an outdoor level surface clear of any debris and effort was made to perform the task in an area away from distracting noise. Before testing, the groin strap was placed on the participant and the smartphone was attached to the strap at the lateral aspect of the participant’s thigh, halfway between the lateral epicondyle and greater trochanter. During dynamic balance testing, participants were instructed to (a) naturally step in place while synchronizing their step timing to the app’s metronome, which lasted for 10 s; and (b) face straight forward during trials (no head-turning). During the practice trial, participants were instructed to raise their knees higher if they were not moving as they normally walked (e.g., feet are not leaving the ground). The pacing metronome was provided for the first 10 s of each trial, 1.15 s per stride. After 10 s, the metronome was turned off, and the participants were instructed to continue stepping for 60 s. The stepping-in-place task was repeated for two trials to enhance reliability [31]. One 30 s practice trial was given to each participant prior to data collection. The participants had their eyes closed for all trials.

### 2.5. Dependent Measures

**Figure 1:**
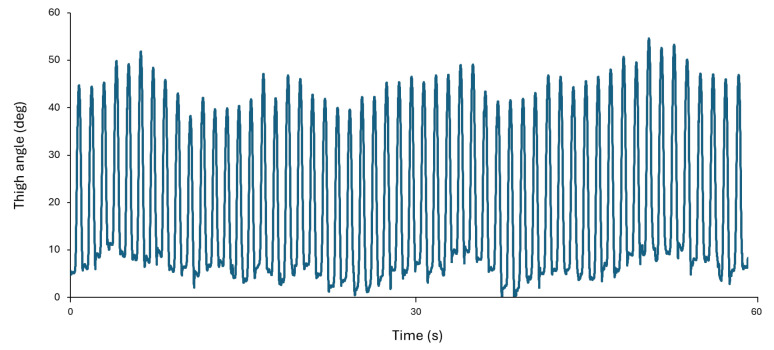
Example time series of thigh-angle data collected from one participant with the AccWalker app.

Neuromotor performance was assessed by examining the thigh flexion angle. The neutral resting position of the leg represented zero degrees. The thigh-angle time series data were obtained from the AccWalker app that used a sensor fusion algorithm fed from accelerometer, gyroscope, and magnetometer data [31]. The extracted thigh-angle data can be visualized in Figure 1 and were processed with a customized MATLAB script (R2022a, MathWorks Inc., Natick, MA, USA). Extracted data were resampled to 100 Hz using spline interpolation. Data were then filtered with a 4th-order low-pass Butterworth filter with a cutoff frequency of 5 Hz. Thigh flexion angles are represented by positive angles, while thigh extension angles are represented by negative angles. The first 10 s for all trials were removed, resulting in the analysis of 60 s rather than 70 s. Kuznetsov et al. [31] demonstrated the reliability and validity of deriving spatial and temporal variables from thigh-angle time series. In this study, we report the coefficient of variation (CV) of peak thigh flexion. Maximum values (peaks) were found for the thigh angle for each stride across the trial. CV was then calculated for peak thigh flex by taking the SD of the peaks divided by the corresponding mean. If participants had two good trials within each condition, their CV was averaged across both trials. Otherwise, the single good trial was used. Only five assessments had only one trial. For the rest, the average of the two trials was used.

### 2.6. Statistical Analysis

Data points for every participant at every time point were not available due to participant dropout, sensor malfunction, etc. Additionally, data points were removed if the Range of Motion (ROM) was less than 10 degrees. All missing data were random. To address the unbalanced sample size and missing data, a Linear Mixed Model (LMM) approach was used to examine the CV of peak thigh flexion. Time (baseline, 6 h, 72 h, 2 weeks, 3 months), group (Exposed: RSOs, SOs, and SO Trainees, and Control: Train Con & Naïve Con) and the interaction between time and group were fixed effects and the individual intercept variance was the random effect. The model specification process was as follows: (a) the original model was tested, if necessary, with a model with different variance structures; (b) the condition x group interaction was omitted if not significant; and (c) the final model was determined based on the results of model comparison with a likelihood ratio test and AIC (Akaike Information Criterion). The results were interpreted based on the output of the final model. The alpha was set at 0.05. Data analysis was performed using R version 4.3.3 [39].

## 3. Results 

### 3.1. Data Processing Results

Prior to analysis, 32 participants who only had baseline measurements and/or withdrew from the study were removed, leaving a total of 123 participants in the exposed group (34 RSOs, 72 SOs, and 17 SO Trainees) and 59 participants in the control group (naïve control = 20, training control = 39), for a total analyzed sample size of N = 182. The demographics and anthropometrics of the included sample are presented in Table 1.

**Figure 2:**
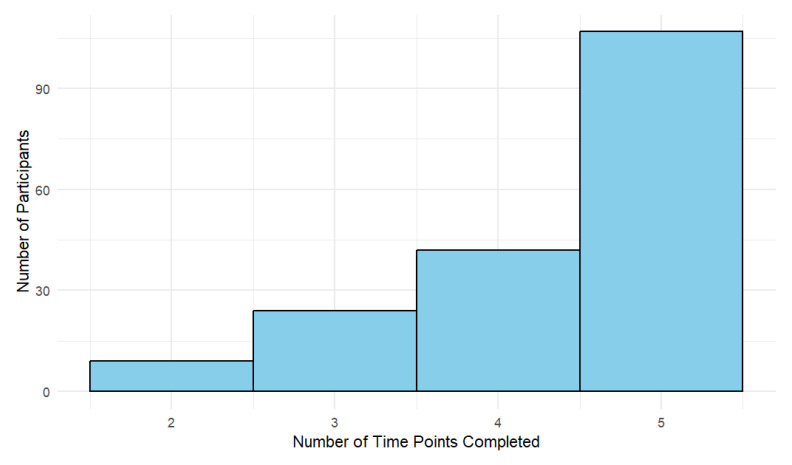
Histogram of the number of participants who completed assessments at 2, 3, 4, or 5 time points.

For the 182 participants’ data that were processed, all had data at two or more of the time points (baseline, 6 h post, 72 h post, 2 weeks post, 3 months post). A histogram of the number of time points completed by the participants is presented in Figure 2, which shows that most participants completed all five time points. The total number of time points represented in Figure 2 is N = 793.

Since two trials at each time point were included in our protocol, the maximum number of trials that could have been included in our analysis was 793 × 2 = 1586 trials. Five of the time points only had one usable trial. Additionally, we applied the Median Absolute Deviation (MAD) approach with a threshold of four to identify outliers based on the distribution of all participants combined for each time point, rather than independently for each participant, which led to the removal of 18 trials. Lastly, 23 trials were removed due to having a ROM of less than 10 degrees. Collectively, the total number of trials included in our analysis was 1586 − 5 (single trials) − 18 (outliers) − 23 (ROM) = 1540 trials. This indicates that only 46 trials out of a possible 1586 trials, or 2.9%, of the data were missing.

Next, various combinations of random effects were injected into an LLM to determine the best fit. We compared two models, (1) one with slope based on site as a random effect as well as the intercept, and (2) one with just the random intercept being individual intercept variance. Based on AIC scores, model 1 (AIC = 2994.1) was a better fit than model two (AIC = 2995.6). After the random intercept was decided, the difference between exposed populations (RSO, SO, and SO trainee) and between training control and naïve control was assessed. No differences were found between populations or between control groups (*p* > 0.05). Based on these results, it was decided to combine the exposed populations into one group (exposed) and to combine both control groups into one control (control). After collating populations and control groups, combinations of random effects were tested again (site and population) to determine the best-fit model. Model 1 consisted of just the individual intercept variance as a random effect, while model 2 included individual variance and site variance as an intercept. Based on AIC scores, model 1 (AIC = 2994.5) was used as the final model, due to it being a better fit than model 2 (AIC = 2996.5). The final LLM model analyzed the difference between group (Exposed vs. Control) against time (5 time points) with the lmer() function from the lem4 package in R statistical software version 4.3.3.

### 3.2. CV Peak Thigh Flexion

**Figure 3:**
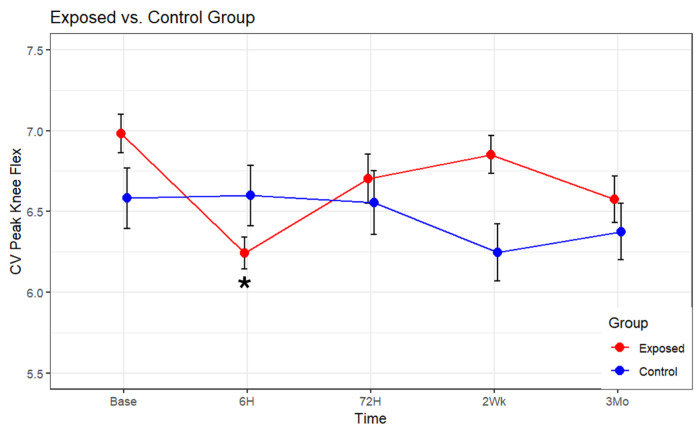
Estimated marginal mean and standard error (SE) bars of CV (%) of peak thigh flexion across time for the exposed and control groups. Asterisks indicate a value different from baseline (base).

The results of the final model for the CV of peak thigh flexion are summarized in Table 2. A significant interaction between groups (Exposed vs. Control) was found, F(4, 601.39) = 2.7856, *p* = 0.025. For the exposed group, the time factor analysis showed significance, F(4, 406.52) = 4.224, *p* = 0.002 (Figure 2). A pairwise comparison was conducted using estimated marginal means (EMMs), specifically employing the Kenward–Roger method to adjust the degrees of freedom. To account for multiple comparisons, a Bonferroni correction was applied, which revealed a significant decline (*p* = 0.001) of CV peak thigh flexion from the baseline (M = 7.01, SE = 0.17) to the 6 h time point (M = 6.26, SE = 0.17). CV peak thigh flexion rebounded at the 72 h mark (M = 6.84, SE = 0.17) to a value not different from baseline and remained at baseline levels at the 2-week and 3-month time points. For the control group, no significant differences in CV peak thigh flexion were observed across time (*p* = 0.434) (Figure 3).

## 4. Discussion

The purpose of this paper was to investigate the effects of RSCBE from HWT on neuromotor performance in military personnel utilizing a longitudinal design. Aligned with our hypothesis, we showed that movement variability—assessed by the CV of peak thigh flexion during a stepping-in-place task—was acutely suppressed in blast-exposed individuals after HWT but returned to baseline levels within 72 h. These results are described below in the context of the optimal movement variability hypothesis [28,32].

The optimal movement variability hypothesis framework is an extension to a field of neuromotor research that investigates how changes in variability patterns can be interpreted relative to the health and functional capacity in biological systems [33,34,35,36,37,38]. The roots of the optimal movement variability hypothesis began by investigating changing variability patterns in the presence of injury, aging, and disease, specifically from complex to less complex signals [32]. The backbone of this research is the complexity hypothesis, which states that healthy individuals exert a certain level of variability, which can be described as mathematically complex [40,41,42]. In the presence of injury, aging, or disease, the system shifts toward less complex behavior. While the beginning of this research focused mainly on cardiovascular dynamics, the theoretical concepts have been extended to human movement, inclusive of neuromotor research on balance [43,44,45]. Further, the framework has been extended to be inclusive of bidirectional shifts in variability. That is, a healthy human system resides in the middle of the continuum of “optimal variability”, and a shift in either direction (less complex or more complex) represents a non-optimal variability pattern, which results in a reduced functional capacity [38,46].

In this study, individuals exposed to RSCBE exhibited suppressed neuromotor variability, indicating a shift toward less complex (i.e., more robotic) behavior. This shift is typically considered a less adaptive form of movement, making it more difficult to shift out of the movement pattern if needed. From an operational perspective, it is not ideal for a Special Operator to exhibit robotic neuromotor patterns, as their tasks may require nimble and agile movement—a behavior that may be difficult to exhibit if they are locked into more robotic movement. We postulate that RSCBE reduces the functional degrees of freedom available to the individual. Functional degrees of freedom refers to a concept where coordinated behavior can be constrained, thereby limiting the functional capacity of the system [47]. Consider the analogy of a person wearing a knee brace. The brace is designed to limit movement in a particular direction, which enhances joint stability and reduces the risk of further injury. However, this stability comes at the cost of functional capacity. The restricted movement limits the person’s ability to exhibit a wide range of movement patterns, which may be needed depending on the task. In this context, stability is increased, but at the expense of functional capacity. In the context of neurotrauma from a subconcussive blast—of which many have been documented—there could be a downstream effect of a reduced ability to exhibit a broad range of movement patterns. As an adjustment to this acute neurological insult, the body constrains the movement patterns to a spectrum of more stable options, thereby stabilizing the system and allowing movement to continue, albeit at the cost of functional capacity. Such a framework can not only help describe the acute suppression of neuromotor variability after RSCBE observed in the current study but may also help explain why a more robotic pattern of variability (as measured by a reduced complexity index score) in a balance test was observed after a concussion [48].

It is important to note that our group-level analysis showed a return of neuromotor variability to baseline levels within 72 h after RSCBE. Due to the intervals between the time-point assessments, we are unable to comment on when this return to baseline behavior occurred between the 6 h and 72 h assessment time points (e.g., at the 7th or 71st hour). Nevertheless, the return to baseline behavior is promising and suggests that neuromotor behavior is only acutely affected, at least at the group level. This observation aligns with Tate et al. [5], showing that neurocognitive and biomarker indicators are altered acutely after subconcussive blast exposure but return to baseline levels after training. It is noted that the blast-exposed group exhibited higher neuromotor variability at baseline relative to the control group, which could have primed them for a steeper decline at the 6 h post-blast assessment time point. However, this observation is tempered by the recognition of the 72 h, 2-week, and 3-month values rebounding to levels not different from the baseline value, suggesting that the decline at the 6 h mark is likely a real observation and not due to inflated baseline performance.

The AccWalker app has been previously used to examine neuromotor performance after head trauma [30]. An important distinction between the current study and the Rhea et al. [30] paper is that the previous study focused on participants with a clinically diagnosed concussion, whereas the current paper focuses on participants with subconcussive head trauma. While the neurometabolic cascade of a concussion has been well documented [49,50]—which is related to symptom recovery [51]—such a mapping has not yet occurred for subconcussive head trauma. As such, it is reasonable to not expect a change in neuromotor performance after subconcussive head trauma to simply be of lesser magnitude relative to a concussion, as the neurometabolic cascade affecting neuromotor behavior may be different, leading to a different presentation in neuromotor performance. This is the case when comparing the current results to the results of Rhea et al. [30]. In the current paper, we show a suppression of neuromotor variability in those who received subconcussive head trauma, whereas Rhea et al. [30] showed an increase in neuromotor variability in those with a concussion. Neurometabolic data were not collected in the two studies, so a direct comparison of this nature is not possible. However, one postulation is that subconcussive head trauma alters neurometabolic processes in a way that leads to a reduction in the degrees of freedom in the neuromotor system—which presents as suppressed (i.e., more robotic) behavior—whereas a concussion leads to significantly more disruption of neurometabolic processes and a more unregulated system, leading to the presentation of increased neuromotor variability. Future work should take a deeper dive into the underlying mechanistic differences in behavior resulting from subconcussive and concussive head trauma.

There are a few limitations to our study. First, since this was a field-based study embedded in military training exercises, we could not control what the participants did before or after the training. The extent to which there were individual differences in sleep deprivation, physical fatigue, and cognitive fatigue was not assessed. Second, all training occurred outdoors and weather (extreme hot or cold) could have affected the participants’ performance. We attempted to standardize the testing environment to the extent possible in a field-based study (i.e., all testing was performed on an outdoor level surface clear of any debris and effort was made to perform the task in an area away from distracting noise), but it was not possible to ensure every participant was exposed to the exact same testing environment. As such, this may have contributed to some outlier data that were excluded. Third, this study focused on one metric of neuromotor performance. Fourth, a machine learning approach was not used to identify participants with blast exposure. Fifth, we did not use a gold standard such as MRI to evaluate the effects of blast exposure. Future work should develop study designs that reduce or eliminate these limitations in order to strengthen their findings. Collectively, while these limitations could have affected some of the participants’ performance, their presentation would have been randomized across the sample and is unlikely to influence the group-level effects presented in this paper.

## 5. Conclusions

We showed that 6 h after HWT, individuals exposed to RSCBE presented with a decrease in neuromotor variability, which we postulate could affect their movement functional capacity. However, blast-exposed participants rebounded to baseline levels within 72 h of HWT, showing that the change in neuromotor variability pattern is acute and short-lived in nature.

## Figures and Tables

**Table 1:** Demographics and anthropometrics of the participants.

	N	Age (yrs)	Height (m)	Weight (kg)	Sex	Years of Duty (yrs)
Exposed Group	123	28.0 ± 4.3	1.81 ± 0.06	88.07 ± 7.50	all male	6.2 ± 4.1
Control Group	59	29.1 ± 5.9	1.77 ± 0.07	86.57 ± 9.12	all male	7.8 ± 6.6

**Table 2:** Analysis of variance (ANOVA) results for exposure groups across time.

	Sum Square	Mean Square	Sum DF	Den DF	*F*	*p*
Exposure	1.634	1.634	1	178.61	0.808	0.369
Time	9.638	2.491	4	601.69	1.192	0.313
Interaction	22.518	5.630	4	601.49	2.786	0.025 ^1^

^1^ This table presents the results of the final model. The sources of variation include exposure and time, degrees of freedom (DF), F statistic, and *p*-value indicating significance.

## Data Availability

Data will be made available upon reasonable request to the corresponding author.
